# The profile and causes of death among medical doctors and dental surgeons in Uganda: 1986 to 2016

**DOI:** 10.4314/ahs.v22i3.70

**Published:** 2022-09

**Authors:** Jacinto Amandua, Victoria Masembe, Jackson Amone, Peter Mukasa-Kivunike, Livingstone Makanga, Jackson Orem, Nazarius Mbona Tumwesigye, Sam Kalungi, Herbert Ariaka, Margaret Mungherera, Fred Nyankori, David Mukunya, Fred Ssentongo Katumba

**Affiliations:** 1 Ministry of Health; 2 Uganda Cancer Institute; 3 Department of Epidemiology and Biostatistics, School of Public Health, Makerere University College of Health Sciences; 4 Department of Pathology, Mulago National Referral Hospital; 5 Uganda Heart Institute; 6 Uganda Medical Association (*Author is deceased); 7 Uganda Medical and Dental Practitioners Council; 8 Department of Community and Public Health, Busitema University; 9 Department of Research, Nikao Medical Center, Kampala, Uganda

**Keywords:** Profile and causes of death, medical doctors, dental surgeons, Uganda

## Abstract

**Background:**

The loss of health workers through death is of great importance and interest to the public, media and the medical profession as it has very profound social and professional consequences on the delivery of health services.

**Objective:**

To describe the profile, causes and patterns of death among medical doctors and dental surgeons in Uganda between 1986 and 2016.

**Methods:**

We conducted a retrospective descriptive study of mortality among registered medical doctors and dental surgeons. Information on each case was collected using a standard questionnaire and analysed. Cause of death was determined using pathology reports, and if unavailable, verbal autopsies. We summarized our findings across decades using means and standard deviations, proportions and line graphs as appropriate. Cuzick's test for trend was used to assess crude change in characteristics across the three decades. To estimate the change in deaths across decades adjusted for age and sex, we fit a logistic regression model, and used the margins command with a dy/dx option. All analyses were done in Stata version 14.0 (Stata Corp, College Station, TX).

**Results:**

There were 489 deaths registered between 1986 and 2016. Of these, 59 (12.1%) were female. The mean age at death was 48.8 years (Standard Deviation (SD) 15.1) among male and 40.1 years (SD 12.8) among females. We ascertained the cause of death for 468/489 (95.7%). The most common causes of death were HIV/AIDS (218/468, 46.6%), cancer (68/468, 14.5%), non-communicable diseases (62/48, 13.3%), alcohol related deaths (36, 7.7%), road traffic accidents (34, 7.3%), gunshots (11, 2.4%), among others. After adjusting for age and sex, HIV/AIDs attributable deaths decreased by 33 percentage points between the decade of 1986 to1995 and that of 2006 to 2016 -0.33 (-0.44, -0.21. During the same period, cancer attributable deaths increased by 13 percentage periods 0.13 (0.05,0.20).

**Conclusion:**

The main causes of death were HIV/AIDS, cancer, non-communicable diseases, alcohol-related diseases and road traffic accidents. There was a general downward trend in the HIV/AIDS related deaths and a general upward trend in cancer related deaths. Doctors should be targeted for preventive and support services especially for both communicable and non-communicable diseases.

## Background

The doctor patient ratio in Uganda is 1:11,000, which is much lower than the WHO recommended ratio of 1:1,000. Attrition of health workers through death is of great importance and interest to the public, media and the medical profession as it has very profound social and professional consequences on the delivery of health services^1^,^2^.

The main causes of attrition of doctors are retirement, change of profession or activity, and migration. However, the risk of violence, illness and death of health workers are other major causes of attrition^3^. Attrition through death is one of the major causes of health worker shortage in Africa which limits the attainment of the health related development goals 4. The supply of doctors per capita may have a direct inverse relationship with the overall mortality in the general population; a higher doctor supply may be associated with a lower mortality rate and improved health outcomes 5, 6. The high mortality of Ugandan medical doctors and dental surgeons may negatively affect the health services in Uganda as it reduces the proportion of doctors per population 3.

Doctors are a category of health workers highly knowledgeable about risk factors for the majority of disease conditions that afflict communities in their environment, how to mitigate the risks, the best treatment options for the various diseases and how to live positively even in situations where one is afflicted with chronic debilitating illnesses. It would therefore be expected that doctors would have a higher life expectancy compared to the general population and would not usually die of preventable conditions. However, it has been asserted that doctors do not benefit from their medical knowledge to improve their health status 7, 8.

Globally, the leading causes of mortality are cardiovascular diseases, cancer, respiratory diseases and road traffic accidents^9^. In Africa, HIV/AIDS, malaria, tuberculosis, perinatal conditions are major contributors to mortality^10^. Most of these disase conditions are preventable, treatable and tend not to have severe long term sequel if managed well. Attrition of health workers due to HIV/AIDS will weaken the health system which will eventually undermine any meaningful health interventions if effective control measures among health workers is not taken^11^.

The high income countries have keenly followed mortality patterns in the population including the health workers for a long time. In 1880s in the UK, the mortality among doctors was worse than all other professions (lawyers and teachers), commercial travellers and miners. The doctors unfortunately even fared worse than innkeepers, butchers, brewers, quarrymen and cutters. The commonest causes of mortality among the doctors in UK during that period were infections (scarlet fever, typhus, diphtheria, typhoid fever, and smallpox), alcoholism, liver disease and suicide^1^. In the UK in the 1970s, the commonest causes of mortality among medical doctors had changed to coronary heart disease, stroke, smoking related lung cancer, accidents, poisoning, suicide and cirrhosis of the liver^12^–^14^.

The life expectancy of medical doctors has improved in high income countries over the last three decades. The risk of all-cause and cause-specific mortality of doctors was found to be lower than those of the general population in many countries such as Denmark, Finland, Norway, USA, France, South Korea and Taiwan^8^, ^15^–^23^. However, suicide rates among medical doctors are higher than in the other professions such as lawyers, teachers, the clergy and the general population^24^–^26^.

The study of the causes of death among health workers has not received much attention in Uganda. A study by Jaggwe in 1987 revealed that the commonest causes of death among 55 Ugandan African doctors were violence, hypertension, HIV/AIDS, cancer, alcoholism, liver cirrhosis, poisoning and stroke 27. A follow up study of a cohort of doctors who graduated from Makerere University Kampala in 1984 showed that by 2004, 22 out of 58 (38%) had died mostly from HIV/AIDS, suicide, one each from road traffic accidents, hepatitis, and alcohol related disease^28^.

There have been a few published studies on the causes of death among doctors in Uganda^27^. Attrition of health workers through death is irreplaceable for the individual staff as they are removed from the available pool of health workers for good. This paper reports the profile and causes of deaths among medical doctors and dental surgeons for the last 30 years so as to inform policy and professional matters for the health and wellbeing of the doctors. This is the largest study on the mortality of medical doctors and dental surgeons in Uganda to date.

## Methods

### Study design

This is a retrospective descriptive study of the profile and causes of death among medical doctors and dental surgeons in Uganda.

### Study population

The study population was all medical doctors and dental surgeons who are in the register of the Uganda Medical and Dental Practitioners Council from 1^st^ January 1986 to 31^st^ December 2016. A list of all doctors who have died between January 1986 and December 2016 was obtained from the Uganda Medical and Dental Practitioners Council and constituted our study samples. We also included doctors who were working outside the country.

Information was also obtained from the districts, hospitals and fellow health professionals on the deaths of any of their colleagues. The hospital directors of the referral hospitals helped to compile the information for their respective catchment areas. Fellow doctors were asked to report any doctors who had died. Additional data was obtained from the hospitals, districts, fellow doctors and families and friends of the deceased doctors and dental surgeons through snowballing. Information was also obtained from the working stations of the doctors and dental surgeons including universities, institutes and private hospitals and clinics. Messages were sent on the UMAchat, an e-forum of the Uganda Medical Association, to avail any information on departed colleagues and verify information on any reported deaths. Information was verified with the Uganda Medical and Dental Practitioners Council.

Where a death was established a questionnaire was completed by research team to capture information on the age, sex, tribe, employment status, place and cause of death. Information was obtained from the Department of Pathology, Mulago National Referral Hospital on the available postmortem reports on the deceased staff. Where the cause of death was not clearly stated, a verbal autopsy was done including contacting relatives and the attending health professionals. The available personal and registration files of the deceased staff in the Ministry of Health, Uganda Medical and Dental Practitioners Council, Mulago National Referral Hospitals and hospitals were also examined to confirm the biodata. Non-communicable disease attributable deaths were classified as deaths due to diabetes mellitus, hypertension, myocardial infarction, renal failure, cerebral vascular accidents, heart failure, cardiomyopathy, and pulmonary embolism. All deaths that occurred during the study period for whom data were available were included in this study. We summarized our findings across decades using means and standard deviations or proportions as appropriate. Cuzick's tests for trends to assess crude changes in characteristics across the three decades 29. To estimate the change in deaths across decades adjusted for age and sex, we fit a logistic regression model, and used the margins with a dy/dx option. We analysed only available results and did not conduct any imputations. This explains the differences in denominators for the various analyses. All analyses were done in Stata version 14.0 (Stata Corp, College Station, TX). Ethical approval was obtained from Mulago Hospital Ethical Review Board.

## Results

### Participant characteristics

There were 489 deaths registered between 1986 and 2016. Of these, 59 (12.1%) were female. The mean age at death was 48.8 years (Standard Deviation (SD) 15.1) among male and 40.1 years (SD 12.8) among females (Table 1). Ethnicity of the doctors included: Ganda (154/485, 31.8%), Ankole (45/485, 9.3%), Soga (41/485, 8.5%), Kiga (39/485, 8.0%), Iteso (36/485, 7.4%), Lango (33/485, 6.8%), Acholi (24/485, 5.0%), Nyoro (15/485, 3.1%), Gishu (12/485, 2.5%) among others. Ranks of the doctors included: medical officers (316/484, 65.2%), consultants (32/484, 6.6%), senior consultant (44/484, 9.1%), professor (25/484, 5.2%), dental surgeons (13/484, 2.7%), senior-lecturer (8/484, 1.7%), among others.

**Table 1:** Participant characteristics overall and by decade of death

Characteristic	Overall N=489	1986–1995	1996–2005	2006–2016	P value*
Age, mean (SD)	47.8 (15.1)	40.5 (13.4)	46.2 (13.6)	55.4 (14.5)	<0.001
Age category, n (%)					
<30	31 (7.0)	14 (10.5)	10 (6.6)	7 (4.4)	
30–39	121 (27.3)	67 (50.4)	40 (26.5)	14 (8.8)	
40–49	110 (24.8)	26 (20.0)	51 (33.8)	33 (20.6)	<0.001
50–59	64 (14.4)	5 (3.8)	21 (13.9)	38 (23.8)	
>=60	118 (26.6)	21 (15.8)	29 (19.2)	68 (42.5)	
Sex					
Female, n (%)	54 (12.0)	13 (9.5)	17 (11.2)	24 (14.7)	0.159
Male, n (%)	398 (88.1)	124 (90.5)	135 (88.8)	139 (85.3)	

### Causes of death

We ascertained the cause of death for 468/489 (95.7%). The causes of death were HIV/AIDS (218/468, 46.6%), cancer (68/468, 14.5%), alcohol related deaths (36/468, 7.7%), road traffic accidents (34/468, 7.3%), myocardial infarction (20/468, 4.3%), diabetes mellitus (15/468, 3.2%), gunshot wounds (11, 2.4%), cerebral vascular accidents (8/468, 1.7%), pulmonary embolism (7/468, 1.7%), suicide (7/468, 1.5%), renal failure (5/468, 1.1%), drugs (4/468, 0.85%), hypertension (3/468, 0.64%), others (32/468, 6.8%) (Table 2). Other causes of death included Ebola, rheumatic heart disease, sickle cell disease, hepatitis, and homicide among others. The most common cancer was cancer of the prostate (23/68, 33.8%), followed by cancer of the pancreas (8/68, 11.8%), cancer of the colon (6/68, 8.8%), hepatocellular carcinoma (5/68, 7.4%), cancer of stomach (3/68, 4.4%) and cancer of the lung (3/68, 4.4%).

**Table 2:** Causes of death overall and by decade of death

Cause	Overall N (%)	1986–1995 n (%)	1996–2005 n (%)	2006–2016 n (%)	P value*
HIV/AIDs	201 (45.3)	90 (67.2)	75 (49.3)	36 (22.8)	<0.001
Cancer	66 (14.9)	6 (4.5)	15 (9.9)	45 (28.5)	<0.001
Alcohol related deaths	35 (7.9)	11 (8.2)	10 (6.6)	14 (8.9)	0.831
RTA	31 (7.0)	2 (1.5)	21 (13.8)	8 (5.1)	0.335
Myocardial Infarction	20 (4.5)	4 (3.0)	8 (5.3)	8 (5.1)	-
DM	15 (3.4)	1 (0.75)	3 (2.0)	11 (7.0)	0.003
Gunshot wounds	11 (2.5)	7 (5.2)	3 (2.0)	1 (0.63)	-
Cerebral Vascular accident	8 (1.8)	2 (1.5)	1 (0.66)	3 (3.2)	-
Suicide	7 (1.6)	2 (1.5)	3 (2.0)	2 (1.3)	-
Pulmonary Embolism	6 (1.4)	1 (0.75)	2 (1.3)	3 (1.9)	-
Renal Failure	5 (1.1)	1 (0.75)	0 (0.0)	4 (2.5)	-
Hypertension	3 (0.68)	0 (0.0)	0 (0.0)	3 (1.9)	-
Drugs	4 (0.9)	1 (0.75)	2 (1.3)	1 (0.63)	-
Others	32 (7.2)	6 (4.5)	9 (5.9)	6 (4.5)	-

### Trend is deaths across decades

**Figure 1:**
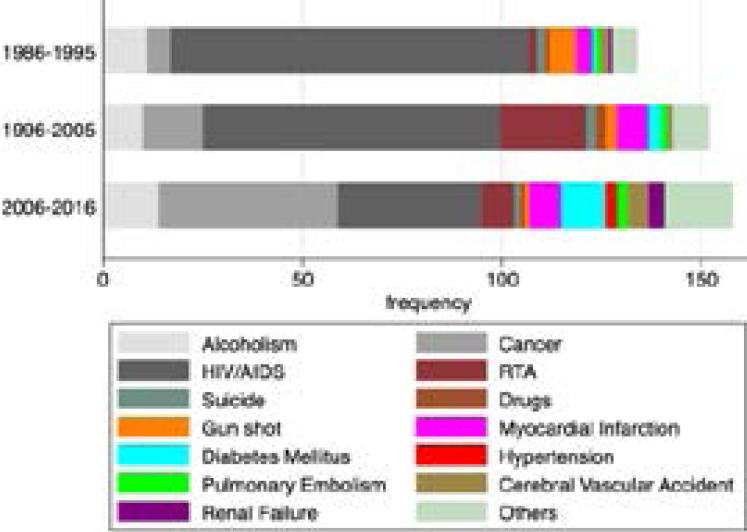
Causes of death among doctors in Uganda between 1986 and 2016

**Figure 2:**
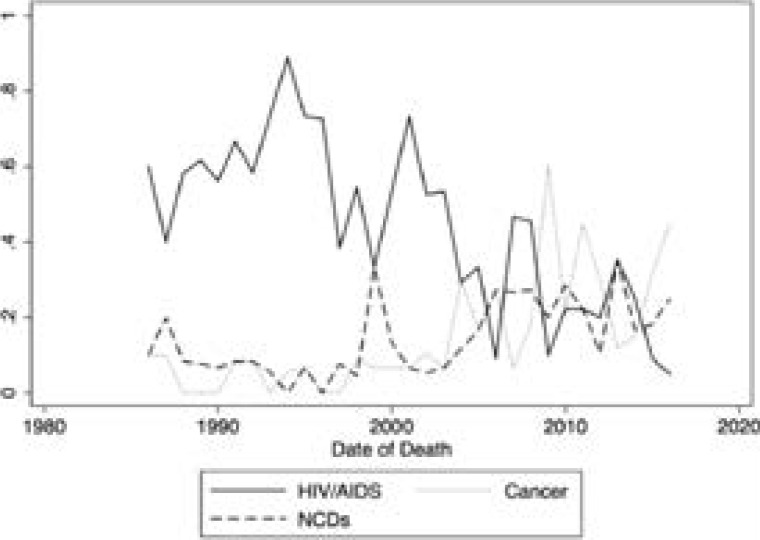
Tread of causes of death among Ugandan doctors between 1986 and 2016

There was a general downward trend in HIV/AIDS related deaths, and an upward trend in the non-communicable diseases and cancer related deaths (Figure 1 and 2). After adjusting for age and sex, HIV/AIDS attributable deaths declined by 13 percentage points between the decade of 1986 to1995 and the decade between 1996 and 2005 (-0.13 (-0.24, -0.01)). During the same period, deaths attributed to NCDs increased by 0.3 percentage points 0.003 (-0.07,0.08), and deaths attributed to cancer increased by 4 percentage points (0.04 (-0.03,0.11)). Upon comparing the decade of 2006 to 2016 to that of 1986 to 1995, HOV/AIDS attributable deaths decreased by 33 percentage points (-0.33 (-0.44, -0.21)), NCD attributable deaths increased by 7 percentage points (0.07 (-0.01,0.15)), and cancer attributable deaths increased by 13 percentage points (0.13 (0.05,0.20)) (Table 3).

**Table 3:** Percentage change in causes of death across decades after adjusting for age and sex

	HIV/AIDs %(95%CI)	P value	NCDs %(95%CI)	P value	Cancer	P value
Decade						
1986–1995	0		0		0	0
1996–2005	–0.13 (–0.24, –0.01)	0.028	0.003 (–0.07,0.08)	0.935	0.04 (–0.03,0.11)	0.261
2006–2016	–0.33 (–0.44, –0.21)	<0.001	0.07 (–0.01,0.15)	0.103	0.13 (0.05,0.20)	0.001

## Discussion

### The level of risk posed by the medical profession to doctors

The medical profession has been a risky occupation all through the times^1^. In Africa, HIV/AIDS and TB are high among the causes of attrition among health workers^30^; in addition to change of working patterns, migration, and ageing. These factors pose serious challenges to the goal of maintaining a sufficient and effective health workforce. The death of health workers leads to higher provider costs; and is also a threat to the quality of care, because it disrupts organizational function, reduce team efficiency, and cause a loss of institutional knowledge.

Health workers in Uganda have a high prevalence of occupational exposure to HIV/AIDS, hepatitis and other blood borne pathogens especially through needle stick injuries, mucosal exposure, and contact with broken skin and cuts with sharp objects^31^. This poses a high risk of transmission of infection to the health workers including doctors.

In the previous study, there were 55 doctors dying over a 32 year period^32^. In this study, there were 489 doctors dying over a 30 year period. This may be due to the premature mortality from HIV/AIDS, road traffic accidents, drugs, alcoholism and Ebola; and the increasing number of medical doctors and dental surgeons qualifying from the medical schools. This is an increase of mortality of about 800% during the last 30 years.

### The major causes of death among doctors working in Uganda

The main causes of death were HIV/AIDS, cancer, road traffic accidents and cardiovascular diseases. It is important to note that HIV/AIDS is the leading cause of mortality among the doctors. Cancer remains high on the list. Taking the incidence of cancer in this country, this may not be unusual. However, the issue of late presentation is also a problem among the doctors. Cancer is one of the main causes of mortality among doctors in high-income countries, and this is becoming the case in Uganda.

Road traffic accidents and myocardial infarction have become a major cause of mortality; however gunshot wounds have reduced. In the Jagwe study, no cases of myocardial infarction were reported 32. In this study, myocardial infarction accounted for 4.51% of the overall mortality and 44% of the cardiovascular diseases. Out of the 24 cases of myocardial infarction, three were in patients with HIV/AIDS and one in a patient with liver cirrhosis. This is a significant increase in the 30 years under study.

The burden of non-communicable diseases is becoming evident as shown in noticeable numbers of diabetes, renal failure, stroke, and hypertension. Renal failure was present in three of the 9 patients with diabetes and one case of HIV/AIDS.

Drug related deaths and suicide are also reported. Of the seven suicide cases, three were in HIV/AIDS patients. The increased risk of suicide and drug abuse among medical doctors has been reported in high-income countries^8^, ^17^, ^25^. There is need for further studies on suicide and drug related health issues among the medical doctors and dental surgeons in Uganda.

The three cases of cardiomyopathy among the doctors is very interesting. The unusual cases included endomyocardial fibrosis, which is often assumed to be a disease of the lower social classes. There is need for further studies on these heart conditions among the doctors.

Globally, mortality from non-communicable diseases is on the increase^33^ and doctors in Uganda are beginning to bear the brunt of these. Therefore, the doctors in Uganda need to take the necessary measures to reduce the risks and consequences of non-communicable diseases and lead more healthy lifestyles.

### Age at death of the medical doctors and dental surgeons

The probability of a Ugandan doctor dying young should be low (1.4 per 1,000) compared to the ordinary Uganda citizen (46 per 1,000) according to the UN estimates^34^. The mean age at death in this study was 48 years. In the Jagwe study, the mean age was 52 years and in a previous study of the mortality of health workers in Arua Hospital the mean age was 40 years^32^, ^35^. The study shows medical doctors and dental surgeons in Uganda now die four years earlier than in the Jagwe series.

For the doctors whose mean age at death were below 50 years, drugs, HIV/AIDS, road traffic accidents, alcoholism, Ebola, guns shots and other violent deaths took a huge toll. In a study of the mortality of young US physicians, the causes were similar and preventable, due to diseases (cancer, atherosclerotic cardiovascular disease, HIV/AIDS), suicide, unintentional injury and homicide^18^.

However for older doctors above 50 years of age; liver diseases, myocardial infarction, hypertension, pulmonary embolism, cancer and diabetes led to the demise of many. The findings are similar to the midlife mortality of Americans which were due to drugs, alcohol, suicide, chronic liver disease and cirrhosis^19^.

The mean age at death increased from 41 years for the period 1986 to 1995, to 47 years for the period 1996 to 2005 and to 55 years for the period 2006 to 2016. Over the three decades, the mean age at death increased by 14 years for the medical doctors and dental surgeons. This is far below the life expectancy at birth in Uganda over the same period. The life expectancy at birth in Uganda has increased from 48 years in 1991, to 50 years in 2002; and to 63 years in 2014 36.

Of the 449 doctors and dental surgeons whose age at death was available 422 (94%) died before the age 75 years, meaning they had premature deaths. With mean age at death of 48 years, each deceased case contributed 27 years of life lost (YLL); and a total years of potential life lost of 11,394 years.

Medical doctors in the UK, USA, Norway, France, Switzerland, Japan and Taiwan generally live longer than the other human service professions such as lawyers and teachers and had lower standardized mortality ratios than the general population^8^, ^17^, ^22^, ^23^, ^37^–^39^. The mean age at death for UK Caucasian doctors is 78 years, UK Asians at 70 years and UAfricans at 62 years^40^. The mean age at death for US physicians was 73 years for Caucasian doctors and 69 years for African doctors^17^. Except for suicide and some violent deaths, the doctors in these countries fare better than their counterparts. The doctors in high income countries are also known to have better lifestyle choices in relation to reduced smoking rates, diet, alcohol moderation and exercise 7, 12–14, 20, 37.

Many medical doctors and dental surgeons in Uganda died from HIV/AIDS, violent deaths including gunshots, homicide, burns and road traffic accidents in the earlier half of the period. This has reduced drastically with the era of ARVs, better security situation and road network. However, as noted, the mortality from non-communicable diseases is increasing including of those on ARVs who now live longer than in the past.

In this study, no proportionate mortality ratios were done as no comparative data was available for the other human service professions. In future studies this should be addressed. One limitation of this study is the lack of data on routine death registration in Uganda.

### Are doctors better protected against preventable killer diseases?

The high mortality of doctors from killer diseases has been known for a long time. During the Ebola epidemics, Ugandan doctors together with the other health professions have been affected more than the general population. The prevalence of hepatitis, HIV and other diseases is known to be high among the health workers 28, 35, 41.

In addition to HIV/AIDS, there was high proportion of mortality from hepatocellular carcinoma, liver cirrhosis, alcohol related deaths, and Ebola among the doctors. For liver cirrhosis, no histology was done to confirm the diagnosis. However, the association between hepatitis and alcohol consumption, both of which are common among medical doctors and dental surgeons, and the liver conditions cannot be ruled out. The social habits of the doctors could have contributed to some of the high proportion of some of the conditions such as HIV/AIDS, liver disease and lung cancer.

All doctors should be orientated regularly on the WHO/ILO guidelines on HIV/AIDS and health services that provide for specific recommendations on prevention, training screening, treatment and confidentiality^42^. The HIV/AIDS epidemic has devastating social, economic and financial effects in Sub-Saharan Africa, including Uganda41. The huge attrition of doctors due to HIV/AIDS is a matter which must be addressed urgently.

No risk analysis per specialty was done in this study. However certain specialties are known to be associated with particular disease conditions such as cancer. Doctors as a group may not be benefitting from their medical knowledge thus exposing them to preventable diseases^7^,^14^. This may explain the high incidence of HIV/AIDS, road traffic accidents, non-communicable diseases, drug related deaths and suicide among the doctors. However, it is known that most preventive interventions do not target the doctors. The non-availability of protective ware may also be a factor. The risk of occupational exposure among doctors is exemplified by the four cases of Ebola which claimed some of the most dedicated doctors in this country. During epidemics such as Ebola, the health workers including doctors have a disproportionate high death rates 43.

Doctors globally are known to be at higher risk for alcoholism, drug addiction and suicide than the other human service professions 1, 7, 8, 23. There seems to be no exception to this in Uganda.

### The impact of the mortality of doctors and dental surgeons on the health system

In Uganda, attrition due to death is a bigger ‘brain drain' among the doctors than emigration since most Ugandan doctors stay to work within the country. The attrition due to mortality is an irreversible outflow of the health workers. This study observed that most of the deceased doctors were young with very bright futures. For those above 50 years, most were in positions of responsibility such as clinical work, teaching, research, politics and opinion leaders.

For a mean age at death of 48 years and retirement age of 60 years, 12 years of active service was lost per doctor. For all the deaths, this amounts loss of 5,844 years of active services. In terms of economic loss this must be huge. However no economic analysis was done. The attrition of doctors due to death will have a negative effect on the attainment of the Sustainable Development Goals (SDGs) and slow the improvement of Socio-Demographic Index (SDI) in Uganda^33^.

Compared to the African American doctors, the Uganda doctors die 20 years younger than their US colleagues. This is a big loss to the health system and the population of Uganda.

## Limitations

The study only analyzed reported cases of deaths. No clinical cases notes were reviewed to confirm the diagnosis and there were very few death certificates available. Most of the information was obtained from the registry and fellow doctors. The registration of deaths and births is very low in Uganda and the Uganda Medical and Dental Practitioners Councils does not keep a routine record of the members who have died. The gazetting of registered medical doctors and dental surgeons has not been regular in the Uganda Gazette making the number of registered practitioners per year difficult to compute. The medical records of the cases were in many instances not complete or available and few post mortems were done to confirm the causes of death. The above factors have limitations on the validity of the findings.

## Conclusion

This study reveals that the main causes of death among doctors in Uganda are HIV/AIDS, cancer, road traffic accidents, cardiovascular diseases and liver conditions. All specialties and ethnic groups are affected. Many of the deaths among the medical doctors and dental surgeons are preventable. Doctors should be targeted for preventive services including those for HIV/AIDS, hepatitis, non-communicable diseases and social factors. Priority should be accorded to the well-being of all health workers in the execution of health services as provided by World Medical Association^44^. Special services to take care of the doctors who need medical assistance should be established; and address the systemic issues that make the doctors unhappy and vulnerable at the workplace and in society^45^.

Further studies are needed to assess the impact of attrition of doctors due to death on the health services and the families of the doctors in Uganda; and the risk factors doctors and dental surgeons are exposed to during their professional duties and social life.
